# An update on the transport and metabolism of iron in *Listeria monocytogenes*: the role of proteins involved in pathogenicity

**DOI:** 10.1007/s10534-015-9849-5

**Published:** 2015-03-28

**Authors:** Justyna Lechowicz, Agata Krawczyk-Balska

**Affiliations:** Department of Applied Microbiology, Faculty of Biology, University of Warsaw, Miecznikowa 1, 02-096 Warsaw, Poland

**Keywords:** *Listeria monocytogenes*, Iron transport, Antilisterial strategies

## Abstract

*Listeria monocytogenes* is a Gram-positive bacterium that causes a rare but severe human disease with high mortality rate. The microorganism is widespread in the natural environment where it shows a saprophytic lifestyle. In the human body it infects many different cell types, where it lives intracellularly, however it may also temporarily live extracellularly. The ability to survive and grow in such diverse niches suggests that this bacterium has a wide range of mechanisms for both the acquisition of various sources of iron and effective management of this microelement. In this review, data about the mechanisms of transport, metabolism and regulation of iron, including recent findings in these areas, are summarized with focus on the importance of these mechanisms for the virulence of *L. monocytogenes*. These data indicate the key role of haem transport and maintenance of intracellular iron homeostasis for the pathogenesis of *L. monocytogenes*. Furthermore, some of the proteins involved in iron homeostasis like Fri and FrvA seem to deserve special attention due to their potential use in the development of new therapeutic antilisterial strategies.

## The importance of iron and its availability for *L. monocytogenes*

Iron is an element absolutely necessary for the functioning of almost all living organisms due to the fact that it plays a key role in such biological processes as oxygen transport, biosynthesis of DNA, energy production and regulation of gene expression. This microelement is a component of cofactors and prosthetic groups of many enzymes such as catalase, nitrogenase, peroxidases, cytochromes and respiratory chain proteins. In physiological conditions, iron can exist in two different oxidation states, i.e. oxidized Fe^3+^ ferric form or reduced Fe^2+^ ferrous form. Both of these forms have different reactivity and different chemical properties. In addition, they can adopt different spin states (low or high), which depends on the type of ligand to which they are bound. All these properties cause that iron is utilized by a large group of enzymes, which explains the great need of living organisms for this microelement. Iron is considered one of the four most important and most abundant compounds that build the Earth’s crust. However, despite such a large amount of iron in the environment, the availability of this microelement for living organisms is low. The reason for this is the low solubility of ferric form predominant in the aerobic environment (Andrews et al. 2003).

The natural environment is not the only ecological niche in which pathogenic organisms, such as *L. monocytogenes*, have limited access to readily available forms of iron. Also, the host organism protects itself against bacterial infections through iron sequestration proteins in the phenomenon called nutritional immunity. This causes the concentration of iron available in the infected organism to be insufficient for the growth of bacteria (Latunde-Dada 2009). In the host organism extracellular iron is bound in the serum by transferrin, and in fluids of the mucous membranes and in the lymph—by lactoferrin. Both these proteins have a high affinity for free iron in the human body. However, in the mammalian organisms, greater than 90 % of iron is present intracellularly, where it is stored in the cytoplasm by another protein-ferritin. In the host organism iron can also be complexed to a tetrapyrrole ring called porphyrin and located in the haem molecule. The most abundant haemoprotein in host organisms is haemoglobin which contains four molecules of haem and is present in erythrocytes (Hammer and Skaar 2011).

*Listeria monocytogenes* is a Gram-positive, facultative human pathogen. This microorganism is widespread in the environment conducting a saprophytic lifestyle in soil, water, waste water, and plant surfaces. Infection occurs by the consumption of food contaminated with *L. monocytogenes*. After ingestion, *L. monocytogenes* is able to enter epithelial cells of the intestinal tract by the interaction of one of several surface proteins with appropriate eukaryotic receptor (Pizarro-Cerda et al. 2012). The most important proteins in the process of bacterial invasion are thought to be internalinA (InlA) and internalinB (InlB). InlA interacts with the human transmembrane glycoprotein receptor E-cadherin, whereas InlB with the hepatocyte growth factor receptor tyrosine kinase c-Met (Mengaud et al. 1996; Shen et al. 2000). In both cases this interaction allows *L. monocytogenes* to invade normally non-phagocytic cell types, including the epithelial cells of the intestinal tract. The interaction of the internalins with appropriate receptor triggers the signalling cascade including ubiquitination of the receptors, recruitment of the endocytosis machinery, the subversion of the phosphoinositide metabolism, and the remodelling of the actin cytoskeleton that lead to the uptake of *L. monocytogenes* by epithelial cells (Pizarro-Cerda et al. 2012). There are a number of virulence factors involved in the pathogenesis process of *L. monocytogenes*, some of which are involved in more than one stage of infection. The expression of the virulence factors is under control of the transcriptional activator PrfA (Chakraborty et al. 1992). After internalisation, the bacteria are able to escape from the phagosomal vacuole owing to the activity of haemolysin (listeriolysin O), a cholesterol-dependent pore-forming cytolysin which acts along with the phospholipases PlcA and PlcB to disrupt the membrane of the vacuole (Bannam and Goldfine 1999; Gründling et al. 2003; Wiśniewski et al. 2006). Upon release from the vacuole, *L. monocytogenes* produces ActA, which promotes nucleation and polymerisation of host cell actin allowing it intracellular movement, spread from cell to cell and to evade the immune system of the host (Domann et al. 1992; Tilney and Portnoy 1989). *L. monocytogenes* is also able to enter mammalian cells owing to listeriolisin O activity which is sufficient to induce bacterial adhesion and subsequent entry into epithelial cells in the absence of InA or InlB signalling (Krawczyk-Balska and Bielecki 2005; Vadia et al. 2011). Furthermore, *L. monocytogenes* may penetrate the host non-specifically and independently of InlA, InlB and haemolysin via M cells overlying the Peyer’s patches in the small intestine of infected individuals (Jensen et al. 1998). The bacteria after traversal of the intestinal barrier migrate with the lymph or blood to the internal organs, i.e. the lymph nodes, liver and spleen (Pron et al. 1998). After proliferation in the liver the bacteria are released into circulation and are capable of infecting many cell types in which they exist intracellularly. *L. monocytogenes* is also able to survive in the extracellular environment of the infected organism, as evidenced by the fact that besides meningitis, encephalitis and perinatal infection one of the common clinical manifestations of listeriosis is septicaemia (Vázquez-Boland et al. 2001). Many years of studies and interest of researchers have made this bacterium the model organism among intracellular pathogens. Given the diversity of niches inhabited by *L. monocytogenes* and the limited availability of iron in these different conditions, it seems obvious that this bacterium must have a wide range of mechanisms for iron acquisition and effective management of this microelement. These mechanisms provide for the survival, growth and proliferation of *L. monocytogenes* in both the natural environment as well as inside and outside the cells of the infected host. The mechanisms of transport of iron and haem of *L. monocytogenes* have been elegantly reviewed by Klebba et al. (2012), while Jesse et al. (2014) have recently reviewed the role of metals in *L. monocytogenes* infections with a focus on the mechanisms that contribute to zinc and copper homeostasis in this organism. This review complements these works and discusses current knowledge of the mechanisms of transport, metabolism and regulation of iron in *L. monocytogenes* with focus on the importance of these mechanisms for the pathogenesis process.

## Iron transport systems in *L. monocytogenes*

The first described system of iron acquisition by *L. monocytogenes* was the citrate-induced system of iron citrate uptake. It has been shown that supplementation of the culture medium with citrate increases the acquisition of [^59^Fe^3+^]—citrate about 200 %, compared to conditions in which no citrate was added to the medium. In addition, these studies show that citrate, which is a ligand of iron, is recognized and bound by a receptor on the surface of the bacterial cells (Adams et al. 1990). This system enables a specific and direct uptake of iron into cells of the microorganism, but so far components of this transport system have not been identified.

Another source of iron used by *L. monocytogenes* are siderophores. Siderophores are iron chelators with low molecular weight (<1000 Da) secreted by various species of bacteria and fungi which have a high affinity for Fe^3+^. Binding of ferric ions by siderophores makes them become soluble, which determines their transport inside the bacterial cell. These compounds are secondary metabolites produced in conditions of iron deficiency in the environment. Hydroxamic or catechol compounds, which are ligands of Fe^3+^ in the siderophores, chelate iron with high efficiency. So far, about 500 siderophores synthesized by various species of microorganisms have been identified, which are classified into different groups according to the type of ligand binding iron present in the siderophore (Andrews et al. 2003). Genes responsible for the biosynthesis of siderophores are absent in the genome of *L. monocytogenes* (Glaser et al. 2001), which confirms also the absence of these compounds in the culture medium (Klebba et al. 2012). Nevertheless, this microorganism has the ability to use as a source of iron siderophores produced by other, coexisting bacterial species, the so-called xenosiderophores (Coulanges et al. 1996; Jin et al. 2006; Simon et al. 1995).

It has been shown that *L. monocytogenes* is able to acquire iron complexed with hydroxamate siderophores such as ferrichrome, ferrichrome A, ferrioxamine B and catechol siderophores including enterobactin and corynebactin (Jin et al. 2006). It is noteworthy that the ability to acquire iron from the different types of siderophores has an adaptive value for *L. monocytogenes*. The ability to acquire iron associated with ferrioxamine B, which is produced by the genus *Streptomyces* commonly found in soil on decaying plant debris, is thought to enable efficient iron acquisition by *L. monocytogenes* in the natural environment. On the other hand, the ability to acquire iron associated with enterobactin seems to be advantageous in the early stages of infection when *L. monocytogenes* is located in the intestines and can use siderophores produced by commensal bacteria. A transport system responsible for the acquisition of iron-enterobactin complexes has not yet been identified. In contrast, an ABC transporter responsible for the transfer of ferric hydroxamate siderophores from the environment into the cytosol of the bacterial cell is already known. This transporter is encoded by the *L. monocytogenes* operon *fhuBCDG* (Jin et al. 2006). The *fhuBCDG* operon includes genes encoding protein FhuD (which has the ability to bind a wide range of hydroxamate siderophores), proteins FhuB and FhuG (which are membrane permeases) and protein FhuC (which is ATP binding protein). The expression of the operon is regulated by Fur (Ferric uptake regulator), which is a global regulator of genes involved in iron metabolism (Jin et al. 2006; Klebba et al. 2012; Xiao et al. 2011). Studies have shown a 90 % reduction of ferrichrome transport across the cytoplasmic membrane in *L. monocytogenes* mutants defective in *fhuC* and *fhuD* genes. These results indicate that the genes of the *fhu* operon encode the primary transport system for hydroxamate siderophores, but the residual ability to transport ferric hydroxamate observed in the mutant strains indicates the existence of a second transport system for these compounds, the significance of which could increase with low concentration of hydroxamate siderophores in the environment (Jin et al. 2006). Such transport redundancy is known in other bacterial species. A single outer membrane protein in Gram-negative species may recognize multiple substrates, for example the Fhu permease system of *E. coli* transports ferrichrome, ferrioxamine B, ferric aerobactin, and ferric rhodotorulate (Rohrbach et al. 1995), or on the other hand, multiple membrane proteins may be receptors for a single compound, for example in *E. coli*, both FepA and FecA are outer membrane proteins able to bind ferric enterobactin complexes (Annamalai et al. 2004; Zhou et al. 1995).

After transportation of iron-siderophore into the cytosol of bacterial cells, the complex must be dissociated to release the iron and to allow its use in metabolic processes. Dissociation of iron from the complexes is most likely coupled with its reduction to the ferrous form, for which siderophores have relatively low affinity (Andrews et al. 2003). The mechanism of intracellular reduction and the enzymes involved in this process have not yet been described in *L. monocytogenes.*

Alternatively, it is postulated that the iron from siderophore complexes can be transported into the cells of *L. monocytogenes* involving a different mechanism being associated with the presence of surface and/or extracellularly localized iron reductase. This enzyme carries out the reduction of insoluble ferric form to a soluble ferrous form (Coulanges et al. 1997; Deneer et al. 1995). It was observed that the reducing activity of the protein is not dependent on the availability of iron in the environment or the growth phase of *L. monocytogenes* culture. It was also shown that the activity of the enzyme was significantly inhibited during growth in anaerobic and low pH conditions. In addition, the reduction process does not occur when the bacterial cells are separated from the iron source by the membrane. This indicates the need for direct physical contact between the cell surface and iron siderophore complexes in order to be able to react (Deneer et al. 1995). Further studies on iron reductase showed that the protein to express its activity requires the presence of NADH, FMN and magnesium ions, which are important cofactors for the transport of a single electron in the reduction reaction (Barchini and Cowart 1996). It is suggested that the presence of cell surface and/or extracellularly localized ferric reductase allows the acquisition of iron compounds such as siderophores, transferrin and neurotransmitters—catecholamine compounds (Brown and Holden 2002). Interestingly, some sources have suggested that the ability of *L. monocytogenes* to use the iron complexed with neurotransmitters such as dopamine, epinephrine and norepinephrine is linked to tropism, which this microorganism shows in relation to cells of the central nervous system. This in turn could explain why encephalitis and meningitis are the most common forms of clinical listeriosis (Coulanges et al. 1997, 1998). After the reduction reaction, it was observed that Fe^2+^ ions were not secreted into the culture medium. Iron-siderophore complexes are therefore considered to be bound by the receptor on the cell surface where the reduction reaction is conducted and the generated Fe^2+^ ions are directly bound and transported into the cytosol (Deneer et al. 1995). The Feo transport system, which is responsible for Fe^2+^ transport in many bacterial species could also be engaged in the transport of the ferrous form of iron. *Escherichia coli* was the first bacterium in which the Feo transport system was identified. It is encoded by the genes *feoA*, *feoB*, and *feoC* (*yhgG*) whose expression is induced under anaerobic conditions and is repressed in the presence of iron in the environment (Cartron et al. 2006). The structure of protein FeoB of *E. coli* comprises two main regions: an N-terminal cytoplasmic domain and a C-terminal polytopic transmembrane domain. Within the N-terminal domain reside the G-protein and the guanine nucleotide dissociation inhibitor (GDI) domains (Eng et al. 2008). The G domain of FeoB is essential for ferrous iron uptake (Marlovits et al. 2002). It is thought that the G domain provides energy for the transport process or that it senses the energy state of the cell and relays this information to the transmembrane domain to regulate transport, whereas the GDI domain stabilizes the binding of GDP to the G domain (Eng et al. 2008). The polytopic transmembrane region of FeoB is proposed to act as a permease for the diffusion of Fe^2+^ into the cell (Marlovits et al. 2002). In the genome of *L. monocytogenes* *feoAB* genes have been identified encoding protein FeoA composed of 75 amino acids and protein FeoB composed of 664 amino acids. *L. monocytogenes* FeoB protein shows 34 % identity and 51 % similarity with the *E. coli* protein FeoB, while the size of these proteins in the two species is identical. A binding sequence for the global regulator Fur has been identified upstream of the *L. monocytogenes feoAB* genes (Jin et al. 2006). The presence of genes encoding system FeoAB in the genome of *L. monocytogenes* suggests that this system may be involved in the transport of Fe^2+^. However, mutation of *feoB* was not observed to have a negative effect on the transport of iron sulfate, which may indicate the existence of an additional Fe^2+^ transport system. Similarly, the absence of a functional protein FeoB had no negative effect on the acquisition of iron complexed with siderophores (Jin et al. 2006).

Recently, it has been proposed that FepB protein is the mysterious ferric reductase of *L. monocytogenes*. FepB is encoded by the last gene of the *fepCAB* operon and is homologous to the iron-dependent peroxidase FepB in *Staphylococcus aureus* and EfeB in *Bacillus subtilis* (Biswas et al. 2009; Miethke et al. 2013; Tiwari et al. 2015). Other genes of this operon encode a putative iron permease FepC and a high-affinity iron-binding lipoprotein FepA. FepB carries a characteristic amino acid motif, including two consecutive arginine residues, which is recognized by a protein export pathway designated the twin-arginine translocation system (Tat). The Tat translocon is a unique system that transports folded protein across the cellular membrane (Berks et al. 2005). In *L. monocytogenes* the Tat system consists of the proteins TatA and TatC, which are encoded by the *tatAC* operon located close to the *fepCAB* operon. The expression of both operons is controlled by the Fur regulator. It was observed that FepB of *L. monocytogenes* is targeted for translocation across the membrane by the Tat system and mutations of *fepB* and *tatC* result in reduced level of ferric reductase activities compared to that of the wild type strain. These observations have led to hypothesize that *fepB* encodes a ferric reductase enzyme. The model for reductive iron uptake has also been proposed in that FepB is translocated across the membrane by Tat onto the cell surface where FepB acts as the ferric reductase enzyme, reducing ferric ions to ferrous ones, which subsequently bind to the iron binding protein FepA, and are then transported by ferrous permease FepC (Tiwari et al. 2015). However, it should be stressed that a number of issues related to the extracellular iron reductase still remain unclear and undoubtedly need further concerted research efforts. First of all, data concerning the physiological analysis of the mutant strains in the genes encoding the individual components of the FepCAB transport system as well as biochemical characterization of the purified proteins are needed to prove the posed hypothesis. This is especially important in view of the potential peroxidase rather than reductase activity of FepB and the observed incomplete reduction of ferric reductase activity in the *tatC* and *fepB* mutants. Moreover, the link between FepCAB and iron siderophore acquisition is missing since the receptor that would be responsible for the binding of iron siderophore complexes and linked to FepB has been not identified.

For pathogenic organisms inside the infected host organism rich sources of iron are haem and haemoglobin (Hammer and Skaar 2011). *Listeria monocytogenes* expresses the protein haemolysin which is able to lyse erythrocytes and thus allows the bacteria access to haemoglobin. In the *L. monocytogenes* genome operon *hupDCG* has been identified, which contains genes encoding an ABC-type transport system allowing the acquisition of haem- and haemoglobin-bound iron. The expression of the *hupDCG* operon is controlled by the Fur regulator (Jin et al. 2006). This system consists of HupD, which is a receptor protein with high substrate specificity, HupG protein which is a membrane permease and HupC which is an ATP-binding protein (Jin et al. 2006; Xiao et al. 2011). In *L. monocytogenes* the process of haem acquisition can proceed in two ways, depending on the concentration of porphyrin in the environment. In the case of relatively high extracellular concentration of haem, acquisition occurs in a sortase-independent manner. It is assumed that under these conditions free haem molecules diffuse through the porous structure of peptidoglycan, are bound by protein HupD anchored to the cytoplasmic membrane and then transported into the cell in a process driven by ATP hydrolysis. The second mechanism is sortase-dependent and takes place when haem is present in the extracellular environment at a concentration below 50 nM. In this case, haem acquisition occurs with the participation of additional proteins which bind haem with high affinity and are anchored in the cell wall by sortase. Sortases are a group of enzymes involved in the covalent binding of secreted proteins to the peptidoglycan of Gram-positive bacteria. Six classes of sortases have been identified so far. The *L. monocytogenes* genome carries genes encoding two of them—sortase A and sortase B. Sortase A cleaves anchored proteins after the threonine in LPXTG whereas Sortase B in the sequence NPXTN. Both enzymes catalyse the formation of a link between the carboxyl group of the threonine and cell wall precursors. In *L. monocytogenes* sortase B (SrtB) is involved indirectly in the process of haem acquisition since it anchors haem-binding proteins, i.e. Hbp1 also called SvpA, and Hbp2 in the cell wall. Expression of Hbp2 and Hbp1 increases under iron limitation conditions. These cell wall proteins are the primary binding site of haem and/or haemoglobin under conditions of low concentrations of haem in the environment. In addition, proteomic analysis showed that a pool of these proteins is secreted into the environment, where they can function as haemophores. After binding of haem, Hbp1 and Hbp2 transfer the porphyrin through the cell wall and deliver it to the HupDCG transport system, which in turn, transports the haem to the cytoplasm (Xiao et al. 2011). Thus, when the environmental haem or haemoglobin concentration is high, the transport takes place only with the participation of the ABC transporter in the cytoplasmic membrane. In contrast, when the concentration of haem drastically decreases additional surface proteins anchored by sortase B are involved, which are the primary haem binding site of the porphyrin, and then transmit it to the ABC transporter. However, it is unclear how haem is extracted from haemoglobin, which is bound by the surface proteins. It is supposed that in this process proteins Hbp1 and Hbp2 are involved and that the mechanism of this process is similar to that identified in *Staphylococcus aureus* (Klebba et al. 2012). In this species, relatively closely related to *L. monocytogenes*, the extraction of haem from haemoglobin is conducted by the sortase-anchored surface proteins IsdA, IsdB and IsdH. These proteins release the porphyrin from haemoglobin, and subsequently transport it to haem-binding proteins with increasing affinity for porphyrin, which interactions lead to the displacement of the haem from the external environment to the cytoplasmic membrane transporter (Hammer and Skaar 2011). Noteworthily, the results of recent research confirm the presumable role of Hbp2 in the process of extraction of haem from haemoglobin (Malmirchegini et al. 2014).

Besides haemoglobin and haem, transferrin may also be another iron source for *L. monocytogenes* in the extracellular environment of the infected organism. It has been shown that *L. monocytogenes* is capable of growth using transferrin as the sole iron source (Hartford et al. 1993). However, the transport system responsible for the usage of this source of iron has not been identified. It is only known that the ability to use iron associated with transferrin depends partially on Fur, since a Δ*fur* mutant strain has reduced ability to use iron complexed to transferrin, compared to the wild-type strain (Jin et al. 2006).

Ferritin is the richest source of iron for *L. monocytogenes* which resides intracellularly in the infected host organism. It was observed that *L. monocytogenes* is able to obtain iron from human ferritin. However, the components of the iron transport system associated with ferritin have not been identified so far (Jin et al. 2006).

**Fig. 1:**
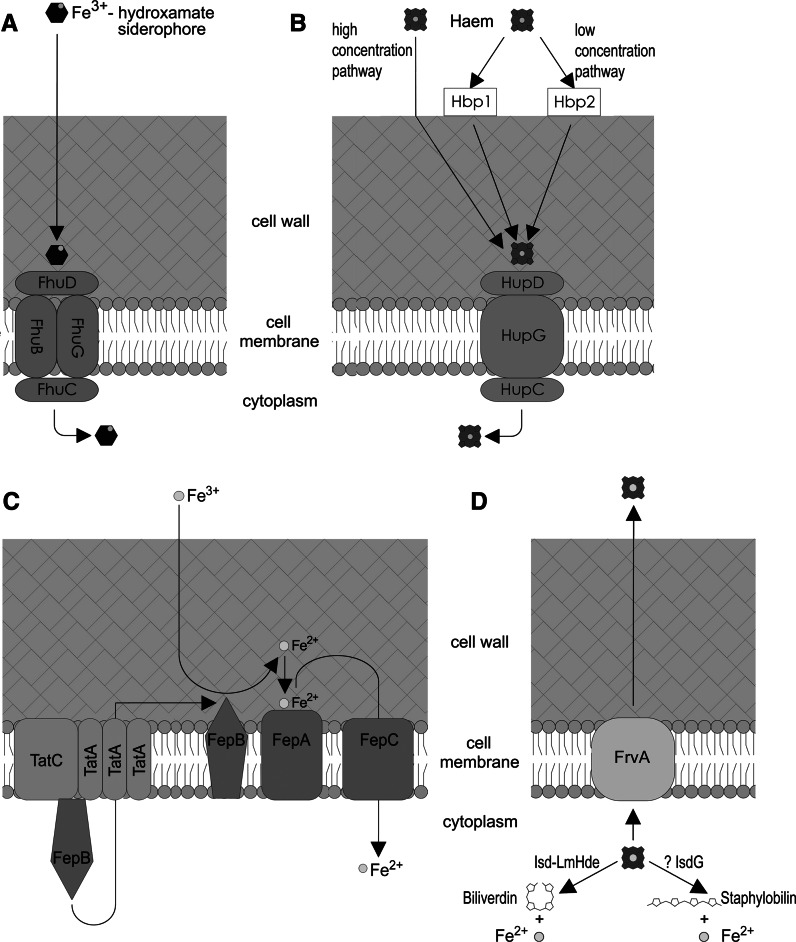
Systems of iron transport in *L. monocytogenes* **a** Transport of hydroxamate siderophores. The transport system consists of FhuD receptor protein, membrane permeases FhuB and FhuG and protein FhuC which is the ATP binding component of the system. **b** Transport of haem. Sortase-independent transport of haem involves the HupD receptor, membrane permease HupG and protein HupC, which is the ATP binding component of the system. Sortase-dependent transport of haem takes place under conditions of low extracellular concentrations of haem (<50 nM). In this case, in addition to proteins HupDCG, the process of haem acquisition involves proteins Hbp1 and Hbp2, which are responsible for capturing porphyrin from the environment. **c** Reductive iron uptake. In the proposed model FepB is translocated across the membrane by Tat translocon. At extracellular surface of membrane FepB acts as the ferric reductase enzyme. After reduction, ferrous ions are bound by the iron binding lipoprotein FepA, and then are transported into the cell by ferrous permease FepC. **d** Export of haem. Haem present in excess is exported to the external environment most probably with the involvement of protein FrvA. Catabolic pathway of exogenous haem in *L. monocytogenes* cells is also shown. Haem acquired from the external environment is degraded by Isd-LmHde enzyme to free iron and biliverdin or, would be degraded by IsgG protein to staphylobilin and Fe^2+^

In summary, despite *L. monocytogenes* being able to use a wide range of compounds as a source of iron, transport systems for only a few of them have so far been identified. The current state of knowledge on transport systems involved in the acquisition of iron is schematically shown in Fig. 1a, b, c.

## The fate of haem after transport into *L. monocytogenes* cell

In conditions of iron limitation in the environment haem, after being transported into the cytoplasm of a bacterial cell, is subjected to degradation by haem oxygenase. In *L. monocytogenes*, this process is catalysed by an enzyme called lsd-LmHde (lsd-type *L. monocytogenes* haem-degrading enzyme), which is encoded by gene *lmo2213*. Structural and functional analysis have shown that the C-terminus of the protein is responsible for the binding of haem, whereas the N-terminal domain determines its catalytic activity. Haem degradation products of the reaction catalysed by haem oxygenases are biliverdin, carbon monoxide (CO) and free iron. Biochemical analyses have revealed that the products of haem degradation catalyzed by Isd-LmHde are also free iron and biliverdin, but the formation of CO has not been observed (Duong et al. 2014). Also present in *L. monocytogenes* is the gene *lmo0484* belonging to the Fur regulon, which shares 32 % identity and 54 % similarity with IsdG, which is a haem-degrading enzyme present in *S. aureus* and other Gram-positive bacteria. IsgG degrades haem to staphylobilin and Fe^2+^ instead of to biliverdin, CO and Fe^2+^ as classical haem oxidases do (Mayfield et al. 2011). Thus, it is possible that *L. monocytogenes* possesses an additional haem-degrading enzyme besides Isd-LmHde. However, this hypothesis needs verification. In conditions of high intracellular concentration, haem can cause severe damage to proteins, lipids, and DNA resulting from the generation of reactive oxygen species (Ascenzi et al. 2005). Therefore, to avoid the potential toxicity of haem, bacteria possess (in addition to haem acquisition systems) also mechanisms enabling the export of porphyrin out of the cell. Such systems have been identified in *Corynebacterium diphtheriae* (Bibb and Schmitt 2010), *S. aureus* (Stauff et al. 2008; Anzaldi and Skaar 2010) and *Lactococcus lactis* (Lechardeur et al. 2012). It is proposed that in *L. monocytogenes* the function of a haem exporter is played by the *frvA* gene product, whose expression is controlled by Fur. It has been shown that a mutation in gene *frvA* increases the sensitivity of *L. monocytogenes* to the toxicity of haemin and haemoglobin. Bioinformatic analyses have shown that protein FrvA has P-type ATPase and hydrolase conserved domains and is homologous to other heavy-metal transporting ATPases in the *Staphylococcus* and *Bacillus* genera (McLaughlin et al. 2012). The fate of exogenous haem in *L. monocytogenes* cells is shown in Fig. 1d.

It is worth mentioning that also a different fate of haem is possible after transport into a bacterial cell. In *S. aureus* which is, as already mentioned, a species closely related to *L. monocytogenes*, when access to iron is not restricted, exogenous haem is sorted intact to the bacterial membrane (Skaar et al. 2004). It is hypothesized that this exogenously acquired haem is destined for proteins involved in respiration since haem is an essential cofactor for proteins involved in the transfer of electrons. This mechanism would enable the restriction of endogenous haem synthesis in cells of *S. aureus*, which in turn decreases the metabolic burden of the bacterium (Hammer and Skaar 2011). It is possible that *L. monocytogenes* also has the ability to incorporate exogenous haem into its proteins, but so far such a phenomenon has been not discovered in this bacterium.

## The intracellular fate of Fe^2+^

Ferrous ions present in the bacterial cell are used directly and indirectly in numerous biological processes—serving as the functional component of cofactors indispensable for the activity of many enzymes. However, high concentrations of a reactive form of iron within the bacterial cell that exhibits aerobic metabolism may be toxic, because hydroxyl radicals are formed in the presence of free Fe^2+^ and hydrogen peroxide by the Fenton reaction (Fe^2+^ + H_2_O_2_ → Fe^3+^ + OH^−^ + OH^•^), which may cause lipid peroxidation, and damage to DNA, protein and other macromolecules (Andrews et al. 2003). This indicates that the fate of iron inside the cell must be subject to strict and precise control. In order to prevent the participation of Fe^2+^ in the Fenton reaction bacteria use the three major groups of proteins sequestering intracellular iron. The first group, bacterioferritins, consists of 24-oligomeric proteins, whose structure contains haem and which are able to store 2000–3000 iron atoms. The second group consists of ferritin, which also has a 24-oligomeric structure and a similar storage capacity to bacterioferritins. However, this group of proteins includes molecules that do not contain haem. The third group of iron storage proteins includes 12-oligomeric Dps (DNA-binding proteins from starved cells) proteins, which do not possess haem and are capable of accommodating 500 iron atoms. The iron storage proteins are composed of identical (or similar) subunits that assemble to form an approximately spherical protein shell surrounding a central cavity that acts as an iron storage reservoir. Ferritins, besides bacteria, are also found in eukaryotes, the bacterioferritins are found only in eubacteria and the smaller Dps proteins are present only in prokaryotes (Andrews et al. 2003). There is significant variability in the type and number of iron storage proteins present in different bacterial species. *E. coli* and *Salmonella enterica* possess two ferritins, one bacterioferritin and a Dps protein (Andrews 1998; Velayudhan et al. 2007), *Campylobacter jejuni* contains one ferritin and a Dps protein (Ishikawa et al. 2003), while *Bacillus subtilis* has two Dps proteins (Chen et al. 1993). Curiously, *L. monocytogenes* produces only a single iron storage protein (Glaser et al. 2001), namely Fri, also called Frm (Mohamed et al. 2006) or Frl (Mohamed et al. 2010), which is in fact a Dps protein (Su et al. 2005). The mechanism of iron sequestration by Dps proteins consists of several stages. First, Fe^2+^ ions translocate into the interior of the dodecamer. The major route of entry of the cations is a N-terminal, negatively charged, hydrophilic pore. Inside the protein cavity iron is bound in the ferroxidase centre located at the two-fold interface between subunits, where two Fe^2+^ ions are oxidized to Fe^3+^. The ferric ions are then moved to the nucleation sites where the process of mineralization of ferric ions takes place, the final product of which is iron hydroxide (FeOOH) (Haikarainen and Papageorgiou 2010). The oxidation of ferrous ions in the ferroxidase centre occurs according to the formula $2 {\text{Fe}}^{ 2+ } + {\text{H}}_{ 2} {\text{O}}_{ 2} + 2 {\text{H}}_{ 2} {\text{O}} \to 2 {\text{FeOOH}} - {\text{P}} + 4 {\text{H}} +$. Dps proteins use H_2_O_2_ as the physiological iron oxidant, which distinguishes them from ferritins and bactoferritins that employ molecular oxygen (Haikarainen and Papageorgiou 2010). Fri of *L. monocytogenes* besides its iron-storage function plays an important role in protection against multiple stresses including oxidative stress, acidification, β-lactam pressure, cold- and heat-shock (Dussurget et al. 2005; Krawczyk-Balska et al. 2012; Krawczyk-Balska and Lipiak 2013; Milecka et al. 2015; Olsen et al. 2005).

Inside the cells iron, apart from being stored, can be bound by proteins in mono- and di-iron reaction centres, can be incorporated into porphyrin rings to form haem, and can be also combined with elemental sulphur to form iron–sulphur (Fe–S) centres. Both haem and iron–sulphur clusters (Fe–S) serve as the key coenzymes of many proteins involved in processes related to metabolism, electron transport, RNA modification and control of gene expression.

Haem biosynthesis is a multi-step, multi-enzyme process that is complicated in bacteria by the absence of some expected enzymes and variability in others (Panek and O’Brian 2002). In *L. monocytogenes* haem biosynthesis enzymes are encoded by *hemA (gtrA),B,C,D,E,H,L,N,Y* genes, from which *hemA(gtrA),C,D,B,L* and *hemE,H* are clustered into 2 operons distantly located in the chromosome, whereas *hemY* is the first gene of an operon containing two other genes i.e. *acpS* (*lmo0885*) and *dal* (*lmo0886*) and *hemN* is transcribed as a monocistronic product (Toledo-Arana et al. 2009). However, the presence of other enzymes involved in haem biosynthesis cannot be excluded since genes encoding for proteins with high homology to bacterial enzymes of haem biosynthesis are present in the *L. monocytogenes* genome, such as *lmo2113* encoding for a protein with high homology to HemQ of *Bacillus subtilis* (Dailey et al. 2010).

Fe–S clusters are formed from ferrous ions and sulphur anions derived from l-cysteine. The formation of Fe–S clusters in bacteria depends on three distinct and highly conserved protein machineries. The first machinery to be discovered, the nitrogen fixation system, is exclusive to the Fe–S cluster assembly of nitrogenase, which converts N_2_ into NH_3_. The second system is termed Isc (iron–sulphur cluster), and the third Fe–S cluster synthesis machinery is designated Suf (sulphur mobilization). The phylogenetic distribution of these three systems is complex. For example, in cyanobacteria the Suf pathway appears to be the major system for Fe–S cluster assembly compared to the Isc pathway. In *E. coli* the relative importance of Suf and Isc is reversed—Isc is responsible for most of the cellular Fe–S proteins and, as such, performs housekeeping Fe–S biosynthesis while Suf performs similar functions to the Isc system, although specifically under iron depletion and oxidative stress. Furthermore, organisms such as *Mycobacterium tuberculosis*, as well as some archaea, appear to possess only the Suf pathway for cluster assembly (Ayala-Castro et al. 2008; Johnson et al. 2005). Likewise, in *L. monocytogenes* the Suf system is the sole pathway for the biosynthesis of Fe–S clusters and is encoded by *lmo2411*-*lmo2415* genes which are homologues for *sufCDSUB* present in other Gram-positive genera (Riboldi et al. 2009).

**Fig. 2:**
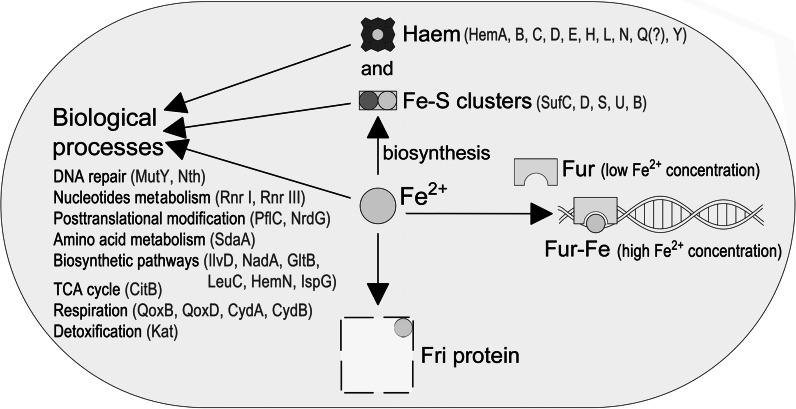
Usage of ferrous iron within *L. monocytogenes* cells. Fe^2+^ ions are primarily used in biological processes, either directly or as a component of haem or iron–sulphur clusters, which act as cofactors of many proteins. Proteins involved in the biosynthesis of haem and Fe–S clusters are given in parentheses. Physiological processes involving iron are given along with examples of engaged proteins. Fe^2+^ ions may also be stored in the single iron storage protein of *L. monocytogenes*, i.e. the ferritin-like protein Fri. Furthermore, Fe^2+^ ions can form complexes with the Fur regulator participating in this way indirectly in the regulation of the expression of genes involved in the transport and metabolism of iron

Fe^2+^ ions present in the cell participate also indirectly in the regulation of the expression of genes engaged in the acquisition and metabolism of iron. This regulation involves the global regulator Fur which forms complexes with iron and binds to a specific DNA sequence (so-called fur-box) in conditions of unrestricted availability of iron. Fur-boxes are located upstream of the gene undergoing regulation. Binding of Fur regulator represses the expression of these genes. However, there are reports that Fur can also act as a negative regulator without binding iron ions and that it can function as an activator (Escolar et al. 1999; Andrews et al. 2003; Troxell and Hassan 2013). Possible modes of the management of iron inside the *L. monocytogenes* cell are schematically shown in Fig. 2.

**Fig. 3:**
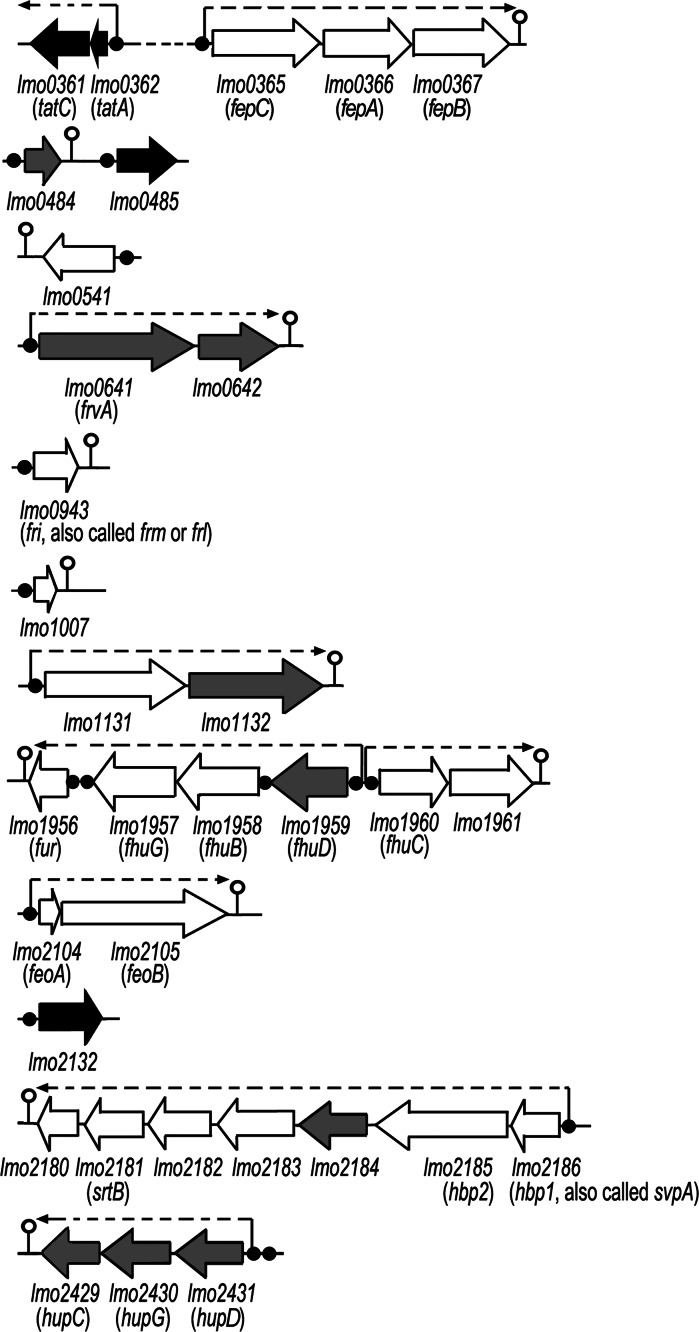
Regulon Fur of *L. monocytogenes*. Genetic organization of Fur regulated genes at 12 chromosomal loci. All genes are drawn approximately to scale using the *L. monocytogenes* EGDe genome sequence data. Loci numbers refer to the National Centre for Biotechnology Information annotation scheme. Genes in *red* indicate those identified exclusively in the study of McLaughlin et al. (2012), *black ones* indicate those identified exclusively in the study of Ledala et al. (2010), and *white ones* indicate those identified in both studies. Fur boxes are represented by *black circles*. *Lollipops* and *dotted arrows* are used to illustrate putative stem loop terminator regions and genes clustered into operons, respectively. (Color figure online)

Recently, the Fur regulon of *L. monocytogenes* has been subjected to two independent genome-wide studies. First, DNA microarray comparative analysis of gene expression changes in a Δ*fur* mutant and wild-type strain in response to iron limitation was examined. This approach allowed the identification of 24 genes regulated by Fur under iron limitation conditions of which 14 were negatively regulated directly by Fur, including mostly genes encoding iron transporters (Ledala et al. 2010). In the second approach a genome-wide search for putative Fur-box consensus sequences in the genome of *L. monocytogenes* using the classical 19 bp Fur-binding motif defined in *B. subtilis* was performed. This led to the identification of 29 putative Fur-regulated loci whose regulation by Fur was further confirmed through comparative RT-PCR transcription analysis in wild-type and a Δ*fur* mutant strain. The identified genes include *hupDCG,* *fhuBCDG* and *fepCAB*. This group also includes genes encoding proteins Fri, sortase B, FeoA, FeoB and proteins of unknown function as well as some genes which have not yet been identified through microarray analysis (McLaughlin et al. 2012). The genetic organisation and characteristic of genes belonging to Fur regulon of *L. monocytogenes* are presented in Fig. 3 and Table 1, respectively.

**Table 1:** Characteristic of the genes belonging to Fur regulon of *L. monocytogenes*

Gene	Function/putative function of encoded protein^a^	Degree of Fur/ Fe control^b^	Match and localisation of Fur site consensus^d^
*lmo0361* (*tatC*)	Sec-independent protein secretion pathway, twin arginine translocase component C (TatC)	22.75/3.61	NA
*lmo0362* (*tatA*)	Sec-independent protein secretion pathway, twin arginine translocase component A (TatA)	19.75/4.29	**GATAATGATAATCATT**t**TC** −25
*lmo0365* (*fepC*)	Putative FTR1 family high-affinity Fe^2+^/Pb^2+^ permease (FepC)	25.03/5.58	**GATAATGATAATCATT**t**TC** −26
*lmo0366* (*fepA*)	Putative lipoprotein involved in iron transport (FepA)	27.61/5.47	NA
*lmo0367* (*fepB*)	Putative Dyp-type peroxidase (FepB)	25.83/4.07	NA
*lmo0484*	Haem-degrading monooxygenase IsdG	5.03/–	**GA**c**A**t**TGA**g**AATCATTATC** −63
*lmo0485*	Hypothetical protein/putative nitroreductase from NADH oxidase and arsenite oxidase family	3.83/2.18	**GATAA**c**G**t**TTATCATT**taa −14
*lmo0541*	ABC transporter substrate-binding protein/putative ABC-type Fe^3+^-hydroxamate transport system	7.01/2.62	**GATAATGA**a**AATCATT**t**TC** −21
*lmo064* (*frvA*)	Heavy metal-transporting ATPase—haem exporter FrvA	−5.64^c^/–	**G**g**TAATG**gg**AATCATTATC** −21
*lmo0642*	Hypothetical protein with unknown putative function	–/–	NA
*lmo0943* (*fri or frm or frl*)	Non-haem iron-binding ferritin, DPS protein	2.4/2.35	at**TAA**g**GATAATCATTAT**a −20
*lmo1007*	Hypothetical protein with unknown putative function	5.61/2.48	g**ATAATGATAATCATT**t**TC** −42
*lmo1131*	ABC transporter ATP-binding protein/putative CydD-like transport system involved in cytochrome bd biosynthesis	9.93/5.97	**GA**c**AATGA**g**AATCATTATC** −159
*lmo1132*	ABC transporter ATP-binding protein/putative MdlB-like multidrug transport system	–/–	NA
*lmo1956* (*fur*)	Ferric uptake regulator Fur	NA/−1.44	**G**ta**A**t**TGATAATCATT**g**T**a −193 **GATAATGAT**g**AT**a**ATT**tag −39
*lmo1957* (*fhuG*)	Ferrichrome ABC transporter permease FhuG	5.86/2.22	NA
*lmo1958* (*fhuB*)	Ferrichrome ABC transporter permease FhuB	4.7/2.01	**G**cg**A**t**TGATAAT**t**ATTATC** −44
*lmo1959* (*fhuD*)	Ferrichrome-binding protein FhuD	–/–	**GA**g**AAT**t**ATTATCA**g**T**ta**C** −14
*lmo1960* (*fhuC*)	Ferrichrome ABC transporter ATP-binding protein FhuC	2.44/2.02	**GA**g**AATGATTATCA**cct**T**a −23
*lmo1961*	oxidoreductase	3.58/2.4	NA
*lmo2104* (*feoA*)	Ferrous iron transport system protein A, FeoA	15.17/7	**GATAATGATTATCAT**gt**TC** −33
*lmo2105* (*feoB*)	Ferrous iron transport system protein B, FeoB	7.76/4.34	NA
*lmo2132*	Hypothetical protein/ putative regulatory protein Crp-like	2.04/2	tt**TA**g**TGATTATC**gc**TAT**a −136
*lmo2429* (*hupC*)	Haem ABC transporter ATP-binding protein HupC	3.03/–	NA
*lmo2430* (*hupG*)	Haem ABC transporter permease HupG	3.12/–	NA
*lmo2431* (*hupD*)	Haem ABC transporter substrate-binding protein HupD	–/–	**GA**a**AA**a**GATTATCA**g**T**cat −156 **GA**a**AAT**a**ATT**c**TCA**a**T**tag −70
*lmo2180*	Hypothetical protein/putative siphovirus Gp157 protein	4.09/4.71	NA
*lmo2181* (*srtB*)	Sortase B, SrtB	12.83/4.6	NA
*lmo2182*	ABC transporter ATP-binding protein /putative ATP-binding component of iron-siderophores, vitamin B12 and hemin transporters and related proteins	13.3/3.97	NA
*lmo2183*	ABC transporter permease/putative permease involved in the uptake of siderophores, haem or vitamin B12	15.76/4.48	NA
*lmo2184*	ABC transporter substrate-binding protein	–/–	NA
*lmo2185* (*hbp2*)	Hemoglobin binding protein 2, Hbp2	8.91/2.99	NA
*lmo2186* (*hbp1* or *svpA*)	Haemoglobin binding protein 1, Hbp1	9.48/4.24	**GA**c**AATGATAATCATTATC** −108

NA, not applicable; ‘–’, no change was observed or data not available

^a^Putative function of the gene is based on annotations provided by NCBI (http://www.ncbi.nlm.nih.gov/gene)

^b^Level of the control is given according to Ledala et al. (2010); Fur control is given as ratios of expression levels in the *fur* mutant (Δ*fur*) in the presence of iron (+Fe) to expression levels in the *L. monocytogenes* wild type (WT) in the presence of iron (Δ*fur* + Fe/WT + Fe) whereas Fe control is given as ratios of expression levels in the *L. monocytogenes* wild type in iron-limiting conditions (−Fe) to expression levels in the *L. monocytogenes* wild type in the presence of iron (WT-Fe/WT + Fe)

^c^The different result was obtained by McLaughlin et al. (2012)

^d^Match is given in comparison to the 19 bp Fur-site consensus (5′GATAATGAT(a/t)ATCATTATC3′) of *L. monocytogenes* defined by McLaughlin et al. (2012), positive matches are in bold and capitalized letters whereas differences in consensus sequence are designated with small letters; localisation is given in relation to translation start site of the gene/operon

## Iron transport and metabolism in *L. monocytogenes*: conclusions and role in pathogenicity

The transport and metabolism of iron in *L. monocytogenes* has been the subject of study for over 20 years. During this time, a lot has been clarified in this matter, especially in relation to the transport of iron. It is now obvious what the gaps in knowledge are. In the pre-genomic era the ability of *L. monocytogenes* to use different sources of iron was extensively studied. In the post-genomic era some of the iron-transport systems were identified, i.e. the FhuBCDG system responsible for the transport of hydroxamate siderophores and the HupDCG system of haem transport with cooperating proteins Hbp1 and Hbp2. Other systems of transports like the system of iron acquisition from human transferrin and ferritin are still awaiting identification and characterization. As already mentioned, also waiting an in-depth analysis is the issue of the proposed role of FepCAB in surface iron reduction and subsequent ferrous ion transport. It is especially intriguing in view of the results of research concerning FepCAB homologs. In *B. subtilis* EfeUOB (YwbLMN) is a homolog of FepCAB. In this system, EfeB (homologous to FepB of *L. monocytogenes*) is a peroxidase that catalyzes ferrous iron oxidation and Fe^3+^ reaction product is transported into the cell by EfeU permease (homologous to FepC of *L. monocytogenes*) (Miethke et al. 2013). In turn, FepB of *S. aureus* (homologous to FepB of *L. monocytogenes*) besides low peroxidase activity has also deferrochelatase activity and therefore is able to extract iron from haem in a manner which preserves the tetrapyrrole ring, generating a free iron atom and protoporphyrin IX (Turlin et al. 2013). Among the genes with unknown function belonging to the Fur regulon Lmo0541 is also worth attention. This protein shares 31 % identity and 49 % similarity with *L. monocytogenes* FhuD. It could thus be assumed that Lmo0541 could be the postulated by (Jin et al. 2006) unidentified second receptor of iron-hydroxamate siderophores. Of interest also seems the aforementioned gene *lmo0484* encoding potential IsdG protein. This suggests that *L. monocytogenes* possesses an additional haem-degrading enzyme besides Isd-LmHde. Undoubtedly, all these hypotheses demand empirical verification.

The existence of a correlation between the uptake and metabolism of iron and the virulence of different bacterial species is well established (Cornelissen and Sparling 1994; Furman et al. 1994; Braun 2005). In the case of *L. monocytogenes* it has also been observed that mutations in the *fur* and *fri* genes reduce the pathogenicity of *L. monocytogenes* in mice indicating that disruption of intracellular iron homeostasis has fatal consequences for the ability of this pathogen to successfully establish infection (Olsen et al. 2005; Newton et al. 2005). In the case of proteins involved in iron transport it has been shown that mutations in genes encoding for the system of haem and/or haemoglobin transport i.e. *hupDGC* lead to 100-fold attenuation of virulence in the mouse model, indicating the importance of this iron source during infection (Jin et al. 2006; Xiao et al. 2011). Likewise, a mutation in *frvA* was shown to drastically diminish virulence properties (McLaughlin et al. 2012), further underlining the crucial role of haem and its management during pathogenesis of *L. monocytogenes*. As could be predicted, among the loci involved in ferric hydroxamate uptake, Δ*fhuD* and Δ*lmo1961* had no effect on virulence (Jin et al. 2006). Surprisingly, attenuation of virulence was not observed in the case of mutants in *hbp1, hbp2* and *srtB* responsible for the acquisition of haem present in low concentrations in the environment (Bierne et al. 2004; Newton et al. 2005). Conflicting reports exist for the importance of the FeoAB transport system in *L. monocytogenes* pathogenesis since no increase in LD_50_ in case of Δ*feoB* was observed (Jin et al. 2006), whereas in another study a significantly lower number of bacteria of Δ*feoB* mutant compared to the wild-type strain in the spleen was detected (McLaughlin et al. 2012). However, this could result from different route of bacteria administration in the studies i.e. intravenous versus intraperitoneal. Interestingly, it was observed that the mutation of the second, putative ferrous iron transport system encoded by operon *fepCAB* had a more pronounced effect on the ability of *L. monocytogenes* to survive in mice than a mutation in *feoB* (McLaughlin et al. 2012) thus putting into question the postulated primary role of the *feoAB* system of *L. monocytogenes* in the transport of ferrous iron.

Listeriosis is a rare, but serious disease, as evidenced by high mortality rate (around 20 %) despite antibiotic therapy (EFSA 2012). The relative ineffectiveness of antibiotic therapy forces to seek other opportunities for the eradication of this pathogen. In relation to this, it is worth mentioning that the link between bacterial proteins involved in the transport and metabolism of iron and virulence makes these proteins promising candidates for targets in vaccine development. The verification of this concept has been initiated in several pathogenic bacteria. For example, the iron sequestering protein IsdB, and iron-uptake ABC transporters have been shown to offer protection against infections caused by *Neisseria gonorrhoeae*, *S. aureus*, and *Streptococcus pneumoniae* (Brown et al. 2001; Cornelissen 2008; Kuklin et al. 2006), whereas ABC iron-transporting proteins were shown to induce an immune response in both *B. anthracis* and *Yersinia pestis* (Gat et al. 2006; Tanabe et al. 2006). Likewise, studies on the identification of the mechanisms of transport and metabolism of iron in *L. monocytogenes* are particularly valuable because they can lead to the development of new strategies against listeriosis. This point of view is supported by the results of recent research. It was shown that administration of antibody targeting the ferritin-like protein prior to infection confers antilisterial resistance in vivo, evidenced in reduced bacterial load and increased survival rates in mouse model of infection (Mohamed et al. 2010). Thus, these results indicate that the ferritin-like protein could be a good candidate for the creation of an anti-*L. monocytogenes* vaccine. More recently, another determinant involved in *L. monocytogenes* haem homeostasis i.e. FrvA has been proved to be even more promising than Fri. Despite significant attenuation in the mouse model of infection, the *frvA* mutant was capable of intracellular growth in antigen-presenting cells. Furthermore, mice immunized with *L. monocytogenes* Δ*frvA* were able to effectively stimulate cellular immunological response at levels comparable with *L. monocytogenes* wild-type strain. Most notably, mice immunized with Δ*frvA*, then subsequently challenged with the wild-type strain, were completely protected from listerial infection (McLaughlin et al. 2013). These results highlight the importance of the protein involved in iron transport and metabolism of *L. monocytogenes* in the development of new therapeutic strategies.
